# Tlx3 Function in the Dorsal Root Ganglion is Pivotal to Itch and Pain Sensations

**DOI:** 10.3389/fnmol.2017.00205

**Published:** 2017-06-28

**Authors:** Chengcheng Huang, Fumin Lu, Ping Li, Cheng Cao, Zijing Liu

**Affiliations:** ^1^Beijing Institute of BiotechnologyBeijing, China; ^2^School of Life Sciences, Anhui Agricultural UniversityHefei, China

**Keywords:** Tlx3, itch, pain, pruriceptor, TRPV1, dorsal root ganglion

## Abstract

Itch, a sensation eliciting a desire to scratch, is distinct from but not completely independent of pain. Inspiring achievements have been made in the characterization of itch-related receptors and neurotransmitters, but the molecular mechanisms controlling the development of pruriceptors remain poorly understood. Here, our RNAseq and *in situ* hybridization data show that the transcription factor Tlx3 is required for the expression of a majority of itch-related molecules in the dorsal root ganglion (DRG). As a result, *Tlx3^F/F^;Nav1.8-cre* mice exhibit significantly attenuated acute and dry skin-induced chronic itch. Furthermore, our study indicates that TRPV1 plays a pivotal role in the chronic itch evoked by dry skin and allergic contact dermatitis (ACD). The mutants also display impaired response to cold and inflammatory pain and elevated response to capsaicin, whereas the responses to acute mechanical, thermal stimuli and neuropathic pain remain normal. In *Tlx3^F/F^;Nav1.8-cre* mice, TRPV1 is derepressed and expands predominantly into IB4^+^ non-peptidergic (NP) neurons. Collectively, our data reveal a molecular mechanism in regulating the development of pruriceptors and controlling itch and pain sensations.

## Introduction

Both pain and itch are crucial sensations warning us away from a variety of harmful stimuli. However, pain and itch evoke distinct experiences and behaviors: pain mainly induces a withdrawal response, while itch elicits a desire to scratch. In contrast to the detailed investigations that have been conducted on pain, the molecular mechanisms of itch are just beginning to be understood. In recent years, marked advances have been made in the characterization of receptors, ion channels, neurotransmitters and neurons in the itch circuits. Several G-protein-coupled receptors (GPCRs) have been reported to participate in itch signal transmission as receptors for pruritogens. The histamine receptors H1R and H4R mediate histamine-dependent itch (Han et al., 2006; Rossbach et al., 2009). Receptors associated with histamine-independent itch include MrgprA3, the receptor for chloroquine, which marks a subpopulation of dorsal root ganglion (DRG) neurons specially linked to itch (Han et al., 2013); MrgprC11, which serves as the receptor for the pruritic agents BAM8-22 and SLIGRL-NH_2_ (Liu et al., 2011; Sikand et al., 2011); and the serotonin receptor HTR7, which regulates acute itch and chronic itch (Morita et al., 2015). In addition to the receptors responding to pruritogens, the neuropeptides gastrin-releasing peptide (GRP) and natriuretic polypeptide b (Nppb) are characterized as vital neurotransmitters specifically relaying itch signals. Genetic ablation of GRPR^+^ neurons or deletion of *Nppb* each resulted in significant reduction of the scratching response to multiple pruritogens, whereas the nociceptive responses remained normal (Sun and Chen, 2007; Sun et al., 2009; Mishra and Hoon, 2013).

Although itch and pain are distinct, they are not completely independent of each other. Some ion channels and receptors respond to both pruritic and painful stimuli. MrgprD is expressed in a unique group of sensory neurons responding to mechanical stimuli and it can also be activated by β–alanine to induce itch (Cavanaugh et al., 2009; Liu et al., 2012). TRP channels are not only responsible for heat/cold and chemical pain but also crucial in transmission of itch signals. H1R-mediated histamine-dependent itch and MrgprA3-/MrgprC11-regulated histamine-independent itch require co-activation of TRPV1 and TRPA1, respectively (Shim et al., 2007; Wilson et al., 2011; Roberson et al., 2013).

Despite the inspiring recent progresses, the mechanisms that control the development of pruriceptors remain largely unknown. The function of the transcription factor Tlx3 in spinal cord was demonstrated to be crucial for the development of GRPR^+^ neurons and its conditional knockout (CKO) in the dorsal spinal cord resulted in attenuated responses to distinct pruritogens (Xu et al., 2013). Our previous study demonstrated that Tlx3 was involved in the differentiation of TrkA-lineage neurons and controlled the expression of a dozen of nociceptive channels and receptors in the developing DRG, including MrgprD, Nav1.9, TRPA1 and so on (Lopes et al., 2012). Some of these molecules are also implicated in sensing itch, thus we speculated that Tlx3 in DRG might also mediate the development of pruriceptors and affect itch-related behaviors. Using the mice with CKO of *Tlx3* in DRG, we demonstrate here that Tlx3 was indeed required for the expression of a majority of known itch-related receptors and neurotransmitters; consequently, *Tlx3cko* mice exhibited markedly attenuated acute and dry skin-induced chronic itch. TRPV1 was vital in the development of chronic itch induced by dry skin and allergic contact dermatitis (ACD). The nociceptive responses to cold, inflammatory pain and capsaicin were also affected in *Tlx3cko* mice. In addition, we found that Tlx3 inhibited the expression of TRPV1 from the perinatal stage and loss of Tlx3 in Nav1.8^+^ neurons led to a substantial expansion of TRPV1, mainly in IB4^+^ non-peptidergic (NP) neurons. Thus, our study reveals a molecular mechanism in regulating the development of pruriceptors.

## Materials and Methods

### Animals

The generation of *Tlx3^F/F^;Nav1.8-Cre* has been described previously (Lopes et al., 2012). Animal experiments were approved by the Animal Care and Use Committee of the Beijing Institute of Biotechnology and were performed in compliance with National Institutes of Health guidelines on the ethical use of animals.

### Behavioral Tests

All the itch and pain behavioral tests were performed on mice approximately 2 months old, with *Tlx3^F/F^* littermates as controls; both males and females were included. All mice were acclimated to the behavior test apparatus for 0.5–1 h on at least three consecutive days before testing. The experimenters were blinded to the genotype of the mice.

### Itch Behavioral Test

Before acclimation, the napes of mice were shaved under anesthesia. Then, the mice were placed in separate boxes for 3 days of acclimation. A camera was positioned to record the behavior and the video was played back for counting. The itch-inducing agents dissolved in 50 μl sterile saline were injected intradermally into the nape, and only the scratching bouts directed towards the injection area were counted for subsequent 30 min. The itch-inducing agents were as follows: Compound48/80 (20 μg, Sigma), Chloroquine (200 μg, Sigma), α-Me-5-HT (30 μg, Tocris), SLIGRL-NH_2_ (100 nmol, Tocris).

For chronic itch, dry skin and ACD models were used. The detailed procedures have been described elsewhere (Zhao et al., 2013). For the dry skin model, mice were treated topically with an acetone/ether mixture followed by water for 7 days, and then spontaneous scratching towards the treated area occurred. For the ACD model, 100 μl 0.15% 2,4-Dinitrofluorobenzene (DNFB) solution was topically applied to the abdominal skin (Day0 sensitization); after Day7, 50 μl 0.15% DNFB solution was applied on the previously shaved nape skin every 2 days until significant spontaneous scratching occurred. The spontaneous scratching bouts were recorded for 1 h in both the dry skin and ACD models. Vehicle or AMG9810 (30 mg/kg, Tocris) was intraperitoneally injected into the mice with obvious spontaneous scratching induced by dry skin or ACD, 10 min later, the spontaneous scratch bouts for subsequent 1 h were recorded.

### Pain Behavioral Test

For Von-Frey test and pinprick, mice were placed in a transparent plastic chamber on an elevated wire grid, and then the plantar surfaces of hindpaws were stimulated with Von-Frey filaments (0.008–1.4 g) or pinprick. A response was considered positive when the mouse quickly withdrew and/or licked its hindpaw. The withdrawal threshold for Von-Frey test was determined as the smallest filament at which at least five positive responses were evoked in ten trials with a 10 s interval. The times of the positive response in ten trials were recorded for pinprick test.

For the hotplate test, mice were placed on a hot plate, and the latency to hindpaw flicking, licking or jumping was recorded. The hotplate test was conducted at three temperatures (50°C, 52°C and 55°C) on two consecutive days; all mice were sequentially tested at each temperature, with an interval of at least 5 min between tests. Cutoff times of 60 s, 45 s and 30 s were used at 50°C, 52°C and 55°C, respectively.

To measure the response to cold, the acetone evaporation and icilin test were used. For acetone evaporation test, mice were placed in a transparent plastic chamber on the wire grid; a drop of acetone was applied on the plantar hindpaw with a tube attached to a syringe. The time that mice spent flicking and/or licking hindpaw after acetone application was recorded. Each mouse was tested twice at a 10 min interval per day on two consecutive days. For icilin test, icilin (30 mg/kg, Sigma) was administered intraperitoneally and numbers of the wet-dog shakes were counted for subsequent 30 min.

To measure capsaicin-induced pain, the plantar hindpaw was injected with capsaicin (2.5 μg/10 μl, Tocris); the duration of hindpaw raising, flicking and/or licking in subsequent 2 min was recorded.

For inflammatory and neuropathic pain test, the Complete Freund’s Adjuvant (CFA) model and the Spared Nerve Injury (SNI) model were used. After the baseline of the mechanical and thermal thresholds were measured, mice were treated with a intraplantar injection of 16 μl CFA or SNI surgery on day 0, subsequent tests were taken on day 2, 3, 6 for CFA model and on day 5, 7, 10, 15, 20 for SNI model. Von-Frey test was used to measure the mechanical threshold (above mentioned). Radiant heat test was taken to measure the thermal threshold. Mice were placed in a transparent plastic chamber on a glass surface, a beam of radiant heat (IITC USA, the radiant intensity parameter AI was set as 25 to result in a baseline latency of 8–10 s for control mice) was focused on the plantar surface of the hindpaw, the latency to hindpaw withdrawing, flicking and/or licking was recorded. Three trials were taken at a 5 min interval. A cutoff time of 20 s was used.

Rotarod test: before testing, mice were trained in a rotarod apparatus at a speed of 5 rpm for several circles (5 min/circles) until the mice did not fall from the rotarod apparatus during the whole 5 min training time. When testing, the rotarod speed was increased gradually from 5 rpm to 40 rpm, the times that mice fell from rotarod were recorded.

### RNA Sequencing

Total RNA of cervical, thoracic and lumbar DRGs from about 2 months old *Tlx3cko* and control mice (*n* = 3 for each genotype) was extracted with an RNeasy Mini Kit (Qiagen) under the manufacturer’s protocol. The RNA samples were sent for RNA sequencing at Beijing Kangpusen Biological Technology Company. General procedures were as follows: after quantification and qualification, a total of 1.5 μg RNA per sample was used as input material for RNA sample preparations. Sequencing libraries were generated using NEBNext^@^Ultra^TM^ RNA library Prep Kit for Illumina (NEB, USA) following the manufacturer’s recommendations and the index codes were added to attribute sequences to each sample. The clustering of index-coded samples was performed on cBot Cluster Generation System using Hiseq 4000 PE Cluster Kit (Illumina). After cluster generation, the library preparations were sequenced on an IlluminaHiseq 4000 platform and 150bp paired-end reads were generated. The RNAseq data was submitted to the GEO database, with accession number GSE93394.

### Reagents

Pruritogens (mentioned above) were dissolved in sterile saline. Capsaicin and AMG9810 (30 mg/kg, Tocris) were dissolved in 7% TWEEN-80 and 3% DMSO/5% TWEEN-80 diluted in sterile saline, respectively. DNFB (0.15%, Sigma) was dissolved in acetone.

### Immunofluorescence (IF) and *In Situ* Hybridization (ISH)

Mice were sacrificed and perfused with ice-cold 4% PFA-PBS (paraformaldehyde dissolved in 1× PBS); the DRGs and spinal cords were dissected, fixed in 4% PFA-PBS (4 h and overnight for IF and ISH, respectively, at 4°C), and then saturated in 20% sucrose overnight at 4°C and embedded in OCT. The detailed procedures for IF and ISH has been described elsewhere (Chen et al., 2006; Liu et al., 2008). For IF, sections were blocked with 5% goat serum plus 0.3% Triton X-100 in 1× PBS for 1 h at room temperature and incubated at 4°C overnight with the diluted first antibodies. Second Day, after washing with 1× PBS, sections were incubated at room temperature for 1 h with the diluted second antibodies. Then the sections from control and *Tlx3cko* mice were mounted and photographed under the confocal microscopy (Zeiss LSM800) with same parameters (laser intensity and voltage). The following antibodies were used: guinea pig anti-TRPV1 (1:1000, Millipore); goat anti-TRPV1 (1:200, Santa Cruz); rabbit and guinea pig anti-Tlx3 (1:2000, a gift from Dr. Carmen Birchmeier, Max Delbruck Center for Molecular Medicine, Berlin, Germany); rabbit anti-peripherin (1:1000, Millipore); rabbit anti-CGRP (1:2000, Peninsula Laboratories); IB4-biotin (1:200, Sigma); rabbit anti-VGLUT1 (1:1000, Synaptic Systems); rabbit anti-c-fos (1:200, Santa Cruz); Alexa 488 goat anti-rabbit (1:1000, Molecular Probes); Alexa 568 goat anti-guinea pig (1:1000, Molecular Probes); Alexa 568 goat anti-rabbit (1:1000, Molecular Probes); Alexa 647 goat anti-rabbit (1:1000, Molecular Probes); Streptavidin-Alexa 488 conjugate (1:1000, Molecular Probes); HRP conjugated donkey anti-goat (1:1000, Santa Cruz) with TSA amplification (PerkinElmer) for TPRV1 antibody from Santa Cruz. For ISH, the detailed procedures and the probes for *TRPV1*, *MrgprA3*, *MrgprC11* have been described elsewhere (Chen et al., 2006; Liu et al., 2008). The probes for *Nppb*, *IL-31ra*, *somatostatin (Sst)*, *GRP* and *GRPR* were amplified with specific primers; cDNA was prepared from adult mouse DRGs and then synthesized *in vitro* with a Digoxigenin (Dig) label (Roche).

For analysis of c-fos expression, mice were anesthetized with pentobarbital, and the left hindpaw was injected with capsaicin (2.5 μg/10 μl) or immersed in a 55°C water bath (30 s, 5 times separated by 1 min intervals), while the right hindpaw remained untreated. Two hours later, mice were sacrificed and perfused with ice-cold 4% PFA-PBS; the L4 to L5-level spinal cords were collected for detection of c-fos expression.

### Statistical Analysis

Data are presented as the mean ± SEM. The statistical graphics and significances were performed on GraphPad Prism5. A paired or unpaired Student’s *t*-test was used for comparison of two groups with *P* < 0.05 considered statistically significant.

## Results

### Decreased Expression of Itch-Related Molecules in *Tlx3cko* Mice

To determine whether Tlx3 can mediate the development of pruriceptors, we used the *Tlx3cko* mouse line constructed through crossing *Tlx3^F/F^* mice with *Nav1.8-Cre* mice. Nav1.8 was selectively expressed in most nociceptive neurons after embryonic day 17 (E17). *Tlx3^F/F^; Nav1.8-Cre* mice (referred to as *Tlx3cko* mice below) specifically lost Tlx3 expression in most TrkA-lineage DRG neurons (Lopes et al., 2012). We performed RNA sequencing (RNAseq) to analyze the transcriptomes in DRGs of both genotypes. The RNAseq results showed that the expression levels of most known itch-related molecules were substantially decreased in *Tlx3cko* DRGs (Table 1). Among them, the histamine receptor H1R (*Hrh1*) mediates histamine-induced itch. The transcriptional expression levels of other histamine receptors were too low to be reliably quantified in our RNAseq results. MrgprA3 is the receptor for chloroquine, and MrgprA3^+^ neurons are specifically linked to itch (Liu et al., 2009; Han et al., 2013); β-alanine can activate MrgprD to evoke a scratching response in mice (Liu et al., 2012). Serotonin (5-HT) can evoke itch via its receptor, 5-HT1f (*Htr1f*). The heterodimeric receptor consisting of IL31ra and Osmr has been reported to mediate itch evoked by interleukin-31 (IL31), which is involved in atopic dermatitis (Dillon et al., 2004; Szegedi et al., 2012). Leukotriene D4 (LTD4) can induce a scratch response in mouse probably due to the activation of its receptor Cysltr2 (Usoskin et al., 2015). The lysophosphatidic acid is closely related to cholestatic pruritus (Kremer et al., 2010), and the expression of its receptors *Lpar3* and *Lpar5* were down-regulated in *Tlx3cko* DRG. PAR-2 and PAR-4 are mainly expressed in the skin and participate in acute and chronic itch evoked by endogenous or exogenous proteases (Reddy et al., 2008, 2010; Soh et al., 2010). These receptors were also detected at low levels in wild-type DRGs, and their expression was markedly down-regulated in *Tlx3cko* mice. Phospholipase C beta 3 (PLCb3) plays a key role in linking GPCRs to their intracellular signaling pathways and mediates itch induced by histamine or serotonin (Imamachi et al., 2009). In addition, the neurotransmitter Nppb is well known to be crucial in relaying itch signals, as *Nppb^−/−^* mice showed strikingly reduced itch responses induced by multiple pruritogens (Mishra and Hoon, 2013). The neuropeptide somatostatin (Sst) marks a subpopulation of itch-sensing neurons in DRG (Stantcheva et al., 2016). In contrast, the expression of *MrgprC11* was significantly increased in *Tlx3cko* mice. Nevertheless, the transcripts of some pruritic receptors, including those of the serotonin receptors *Htr2a*, *Htr3a* and *Htr7*, were not restricted to Nav1.8^+^ nociceptors and remained largely unchanged in mutant DRGs.

**Table 1:** The transcription profiles of itch-related genes in *Tlx3cko* and control dorsal root ganglions (DRGs) by RNAseq.

Gene	Pruritogens	Transcript reads			
		Control	Tlx3cko	Fold change	*P* value
*Hrh1*	Histamine	134	17	0.12	1.34E-20
*Lpar3*	Lysophosphatic acid	1730	473	0.27	0.003247
*Lpar5*		211	30	0.14	0.023825
*MrgprD*	β-alanine	2689	770	0.29	7.96E-05
*MrgprA3*	Chloroquine	273	17	0.06	3.95E-18
*Osmr*	IL31	700	341	0.49	0.002588
*IL31ra*		438	19	0.04	0.013328
*Cysltr2*	LTD4	182	17	0.09	0.004932
*Htr1f*	Serotonin	83	31	0.37	0.004838
*F2rl1(PAR2)*	Mucunain & Cathepsin S & Tryptase	18	0	0	0.032255
*F2rl3(PAR4)*		35	20	0.56	0.036852
*MrgprC11*	BAM8-22 & SLIGRL-NH_2_	229	440	1.92	0.035275
*PLCb3*	Enzyme	13159	8245	0.63	5.56E-06
*Nppb*	Neuropeptide	287	3	0.01	6.07E-59
*Sst*		370	7	0.02	0.001156
*Htr2a*		93	93	1	1
*Htr3a*	Serotonin	2671	2859	1.08	1
*Htr7*		288	342	1.19	0.854365

*The itch related gene symbols were listed in left column. The pruritogens indicate the chemicals activating the channels or receptors encoded by the listed genes, or its property like “enzyme, neuropeptide”. The transcript reads were represented as mean of three independent samples from control and *Tlx3cko* mice. The fold changes were calculated by mean_Tlx3cko_/mean_control_. The *P* values were obtained by an unpaired two tails student’s t-test with *p* < 0.05 considered significant*.

**Figure 1:**
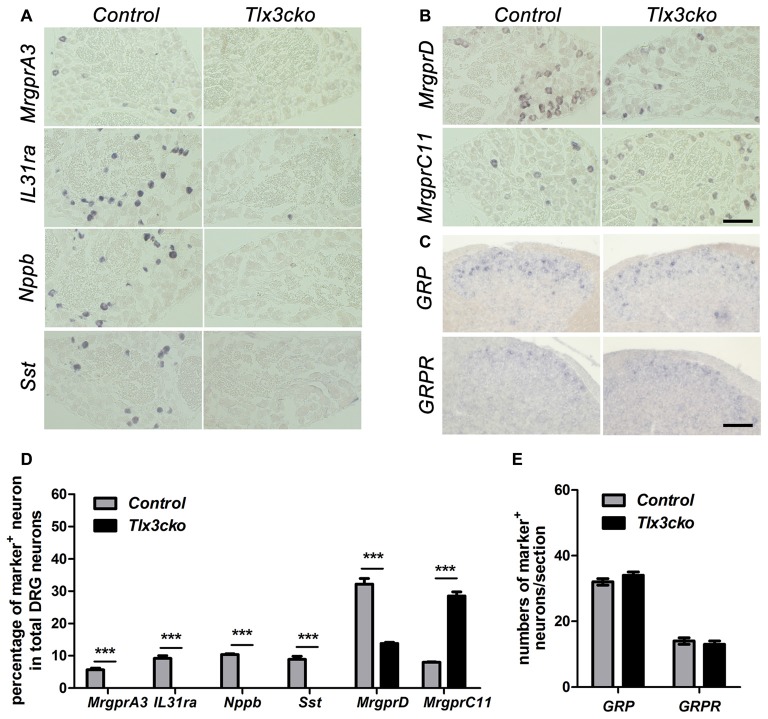
Expression of a subset of itch-related molecules in *Tlx3cko* dorsal root ganglions (DRGs) and spinal cords. *In situ* hybridizations with indicated probes were performed on transverse sections of lumbar DRGs **(A,B)** and spinal cords **(C)** from adult control and *Tlx3cko* mice, the statistical data were shown on **(D,E)**. Compared with that in control mice, expression of *MrgprA3*, *IL31ra*, *Nppb* and *Sst* were almost completely eliminated in *Tlx3cko* mice **(A)**. The percentage of MrgprD^+^ neurons in *Tlx3cko* mice was markedly reduced (control, 32.2% ± 1.7%, *n* = 3; *Tlx3cko*, 13.8% ± 0.4%, *n* = 3; *p* = 0.0004). The percentage of MrgprC11^+^ neurons was greatly increased in *Tlx3cko* mice (control, 8% ± 0.1%, *n* = 3; *Tlx3cko*, 28.5% ± 1.3%, *n* = 3; *p* < 0.0001) **(B)**. **(C)** The numbers of GRP^+^ and GRPR^+^ neurons in the spinal cords of *Tlx3cko* and control mice were comparable (GRP^+^ neruons : control, 32 ± 1/section; *Tlx3cko* 34 ± 1/section; *n* = 28–32 sections from 3 mice, *p* = 0.3753; GRPR^+^ neurons: control, 14 ± 1/section; *Tlx3cko* 13 ± 1/section; *n* = 22–23 sections from 3 mice, *p* = 0.8294). ***Indicates *p* < 0.001, Scale bar represents 100 μm.

To further confirm our RNAseq results, we performed a series of* in situ* hybridization experiments to examine the expression of itch-related receptors and neurotransmitters. Consistent with the RNAseq data, in *Tlx3cko* mice, the expression of *MrgprA3*, *Nppb*, *IL31ra* and *Sst* was completely eliminated (Figures 1A,D), and the percentage of MrgprD^+^ neurons decreased by approximately 57%, while the percentage of MrgprC11^+^ neurons was indeed approximately tripled compared to control littermates (Figures 1B,D). GRP and its receptor GRPR are vital in the transmission of itch signals (Sun and Chen, 2007; Sun et al., 2009). As that no obvious transcript read for *GRP* and *GRPR* was obtained in our DRG RNAseq, consistent with the previous reports that GRP and GRPR are mainly expressed in spinal cord not in DRG (Solorzano et al., 2015), we performed *in situ* hybridization experiments to detect their expression in spinal cords. The numbers of GRP^+^ and GRPR^+^ neurons were similar between *Tlx3cko* and control spinal cords (Figures 1C,E), suggesting that selective knockout of *Tlx3* in Nav1.8^+^ DRG neurons does not affect the development of GRP^+^ and GRPR^+^ neurons located in the superficial dorsal spinal cord. Thus, Tlx3 was required for the expression of multiple itch-related molecules in DRG.

### Attenuated Acute and Dry Skin-Induced Chronic Itch in *Tlx3cko* Mice

**Figure 2:**
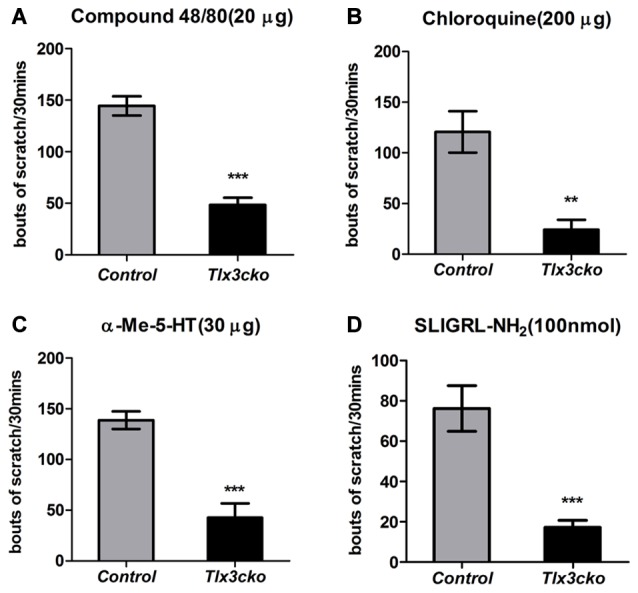
Attenuated acute itch in *Tlx3cko* mice. The acute itch responses evoked by distinct pruritogens were examined in both *Tlx3cko* and control mice **(A–D)**. **(A)** Decreased scratch bouts evoked by Compound48/80 (20 μg) in *Tlx3cko* mice (control, 145 ± 9, *n* = 6; *Tlx3cko*, 48 ± 7, *n* = 6; *p* < 0.0001). **(B)** Decreased scratch bouts evoked by Chloroquine (200 μg) in *Tlx3cko* mice (control, 120 ± 20, *n* = 10; *Tlx3cko*, 24 ± 9, *n* = 6; *p* = 0.0037). **(C)** Decreased scratch bouts evoked by α-Me-5-HT (30 μg) in *Tlx3cko* mice (control, 138 ± 6, *n* = 6; *Tlx3cko*, 42 ± 13, *n* = 6; *p* = 0.0002). **(D)** Decreased scratch bouts evoked by SLIGRL-NH_2_ (100 nmol) in *Tlx3cko* mice (control, 76 ± 11, *n* = 6; *Tlx3cko*, 17 ± 3, *n* = 7; *p* = 0.0003). **, ***Indicate *p* < 0.01, *p* < 0.001, respectively.

To explore whether the deficits of these Tlx3-dependent molecules affected itch-related behaviors, we examined the acute and chronic itch responses in *Tlx3cko* and control mice. We first monitored site-directed acute itch behavior after nape injection of multiple pruritogens in *Tlx3cko* mice and control littermates. Itch can be classified into histamine-dependent itch and histamine-independent itch. Compound 48/80 can induce histamine-dependent itch by activating the mast cells to release histamine (Sugimoto et al., 1998). The nape injection of Compound 48/80 induced a robust scratch response towards the treated area in control mice, while scratching bouts were reduced by approximately 67% in *Tlx3cko* mice (Figure 2A), suggesting that Tlx3 controlled histamine-dependent itch. The anti-malarial drug chloroquine, the serotonin derivative α-Me-5-HT and the peptide SLIGRL-NH_2_ have been reported to elicit histamine-independent itch through their receptors located in pruriceptors (Imamachi et al., 2009; Liu et al., 2009, 2011). In *Tlx3cko* mice, the site-directed scratch responses induced by these pruritogens were all significantly reduced but not completely lost (Figures 2B–D), indicating that Tlx3 also controlled histamine-independent itch. Despite the up-regulation of *MrgprC11* or the continued presence of some serotonin receptors, *Tlx3cko* mice still displayed obvious deficits in itch induced by SLIGRL-NH_2_ and α-Me-5-HT, probably due to the down-regulation of *Nppb* and *PLCb3* which act downstream of the activation of itch-related receptors. Consequently, deficits of multiple itch-related molecules impaired both histamine-dependent and histamine-independent acute itch.

**Figure 3:**
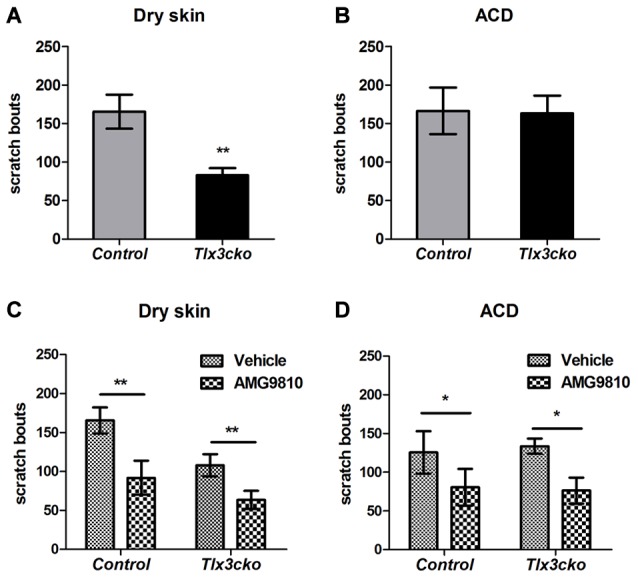
Impaired dry skin-induced chronic itch in *Tlx3cko* mice and the involvement of TRPV1 in chronic itch. The dry skin and allergic contact dermatitis (ACD) models were used to assess the responses to chronic itch. **(A)** Impaired chronic itch induced by dry skin in *Tlx3cko* mice (control, 165 ± 22, *n* = 14; *Tlx3cko*, 83 ± 9, *n* = 9; *p* = 0.0087). **(B)** Similar response to chronic itch induced by ACD in *Tlx3cko* and control mice (control, 166 ± 30, *n* = 7; *Tlx3cko*, 163 ± 23, *n* = 7; *p* = 0.9384). **(C)** TRPV1 antagonist AMG9810 significantly reduced the spontaneous scratching induced by dry skin in both *Tlx3cko* and control mice (control: vehicle 166 ± 17, *n* = 9 vs. AMG9810 92 ± 22, *n* = 9; *p* = 0.0028; *Tlx3cko*: vehicle 108 ± 12, *n* = 5 vs. AMG9810 63 ± 12, *n* = 5; *p* = 0.0031). **(D)** AMG9810 significantly alleviated chronic itch induced by ACD (control: vehicle 125 ± 27, *n* = 7 vs. AMG9810 80 ± 23, *n* = 7; *p* = 0.0305; *Tlx3cko*: vehicle 133 ± 10, *n* = 7 vs. AMG9810 76 ± 17, *n* = 7; *p* = 0.0271). *,** Indicates *p* < 0.05, *p* < 0.01, respectively.

A variety of endogenous pruritogens, such as histamine, serotonin, IL31 and leukotrienes, have been reported to be directly involved in chronic itch, including atopic dermatitis and dry skin diseases (Szegedi et al., 2012; Morita et al., 2015). The receptors for these mediators were dramatically down-regulated in *Tlx3cko* mice, suggesting that Tlx3 might also regulate chronic itch. To explore this hypothesis, we constructed two chronic itch models, dry skin and ACD, which are prevalent symptoms in patients with skin diseases and systemic disorders. In the dry skin model, control mice exhibited robust spontaneous scratching, whereas *Tlx3cko* mice scratched much less (Figure 3A), suggesting impairment of dry skin-induced chronic itch. Unlike in the dry skin model, the numbers of spontaneous scratch bouts were comparable between *Tlx3cko* and control mice in the ACD model (Figure 3B), demonstrating that down-regulation of Tlx3-dependent pruritic molecules was insufficient to disturb ACD-induced chronic itch. Thus, Tlx3 was required for chronic itch induced by dry skin but not by ACD.

Given the dramatically decreased expression of itch-related molecules, the presence of normal ACD-induced itch and partly decreased dry-skin-induced itch in *Tlx3cko* mice suggested that *Tlx3cko* mice still retained alternative molecules to transmit chronic itch input. In addition to its association with histamine-dependent acute itch, increased TRPV1 expression in the skin and DRG has been reported in multiple chronic itch diseases, such as dry skin, atopic dermatitis and prurigo nodularis (Miyamoto et al., 2005; Alenmyr et al., 2009; Yun et al., 2011). To assess whether chronic itch was dependent on the TRPV1 signal pathway, we treated *Tlx3cko* and control mice with the TRPV1 antagonist AMG9810. In mice of either genotype with dry skin or ACD, AMG9810 markedly reduced spontaneous scratch bouts (Figures 3C,D), suggesting that TRPV1 was indeed involved in the development of chronic itch, at least in dry skin and ACD.

### Impaired Response to Cold, Inflammatory Pain and Elevated Response to Capsaicin in *Tlx3cko* Mice

**Figure 4:**
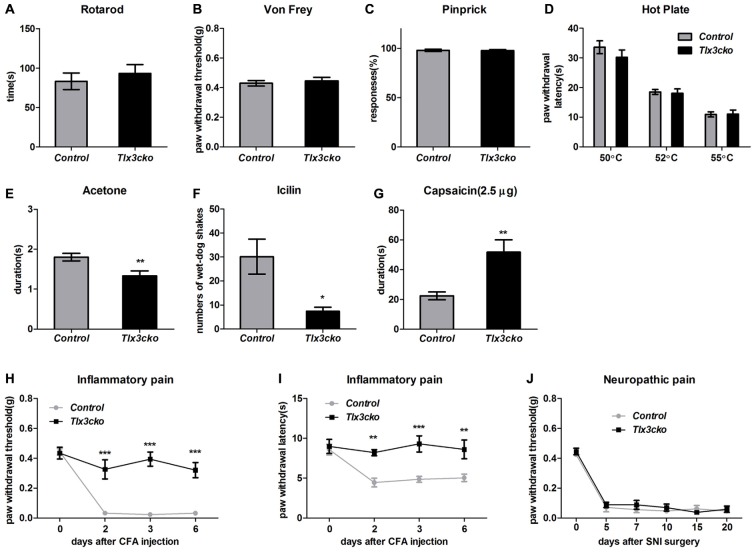
Impaired response to cold, Inflammatory pain and elevated response to capsaicin in *Tlx3cko* mice. **(A)** Rotarod test. *Tlx3cko* mice showed normal sensormotor coordination (fallen time: control, 83.3 ± 10.5 s, *n* = 7; *Tlx3cko*, 93.3 ± 11.2 s, *n* = 7, *p* = 0.5279). Normal mechanical sensitivity in *Tlx3cko* mice tested by Von-Frey filaments (control, 0.42 ± 0.2 g, *n* = 11; *Tlx3cko*, 0.45 ± 0.2 g, *n* = 14; *p* = 0.6073) **(B)** and pinprick (control, 98% ± 1.2%, *n* = 5; *Tlx3cko*, 98% ± 1.0%, *n* = 7; *p* = 0.9298) **(C)**. **(D)** Hotplate assay. *Tlx3cko* mice showed similar thermal thresholds with control mice. For 50°C, control, 33.6 ± 2.1 s, *n* = 8; *Tlx3cko*, 30.2 ± 2.4 s, *n* = 9; *p* = 0.3233. For 52°C, control, 18.5 ± 0.8 s, *n* = 8; *Tlx3cko*, 18.1 ± 1.5 s, *n* = 9; *p* = 0.8244. For 55°C, control, 10.9 ± 0.9 s, *n* = 8; *Tlx3cko*, 11.1 ± 1.3 s, *n* = 9; *p* = 0.9269. Cold pain was impaired in *Tlx3cko* mice examined by acetone evaporation assay (control, 1.8 ± 0.1 s, *n* = 17; *Tlx3cko*, 1.3 ± 0.1 s, *n* = 14; *p* = 0.0048) **(E)** and Icilin injection (control, 30 ± 7, *n* = 6; *Tlx3cko*, 7 ± 2, *n* = 5; *p* = 0.0217) **(F)**. **(G)** Enhanced response to capsaicin in *Tlx3cko* mice (control, 22.3 ± 2.6 s, *n* = 9; *Tlx3cko*, 51.7 ± 8.3 s, *n* = 10; *p* = 0.0052). **(H,I)** Impaired response to inflammatory pain in *Tlx3cko* mice, **(H)** mechanical threshold and **(I)** thermal thresholds (repeated measures two-way ANOVA followed by Bonferroni post test, *p* < 0.01). **(J)** Normal response to neuropathic pain in *Tlx3cko* mice (repeated measures two-way ANOVA followed by Bonferroni post test, *p* > 0.05). *, **, ***indicate *p* < 0.05, *p* < 0.01, *p* < 0.001, respectively.

To exclude the possibility that the significantly reduced scratch responses evoked by distinct pruritogens in *Tlx3cko* mice were resulted from the impaired sensormotor coordination, rotarod test was performed on *Tlx3cko* and control mice. The fallen time was not significantly different between *Tlx3cko* and control littermates, suggesting that the sensormotor coordination of *Tlx3cko* mice was normal (Figure 4A).

Tlx3 was previously reported to regulate the generation of a cohort of nociceptive channels and receptors, it led us to explore whether the pain behaviors were also affected like itch behaviors in *Tlx3cko* mice. Here we carried out a series of behavior tests to assess the responses to acute mechanical, thermal, cold and chemical stimuli as well as chronic inflammatory pain and neuropathic pain in *Tlx3cko* mice.

Von-Frey test and pinprick were used to measure the sensitivity to mechanical stimuli. In Von-Frey tests, *Tlx3cko* mice showed similar mechanical threshold as the control littermates (Figure 4B). Compared with the light mechanical stimuli of the Von-Frey filaments, the mechanical stimuli in pinprick test were intense. We observed largely unchanged response to pinprick (Figure 4C), suggesting that the mechanical threshold was not affected in *Tlx3cko* mice.

We placed the mice on a hotplate to assess the response to noxious heat. The tests were performed at three temperatures (50°C, 52°C and 55°C). The latency of the hindpaw flicking, licking or jumping was no change between *Tlx3cko* and control mice at any test temperature (Figure 4D), suggesting that the response to noxious heat was also unaffected in *Tlx3cko* mice. Next, we used the acetone evaporation and icilin model to assess the response to cold. In acetone evaporation test, *Tlx3cko* mice spent significantly less time licking or raising hindpaw compared to control littermates (Figure 4E). Icilin, a compound activating TRPM8 and TRPA1, is able to induce a cooling sensation and wet-dog shake. Just like that in the acetone evaporation test, *Tlx3cko* mice presented much less wet-dog shakes than control littermates (Figure 4F). These data suggested that the response to cold was impaired in *Tlx3cko* mice.

To assess the response to chemical pain, we injected capsaicin into the plantar of hindpaw. Intraplantar injection of capsaicin elicited markedly enhanced nociceptive responses in *Tlx3cko* mice, indicated by that the mutant mice spent significantly more time raising, flicking or licking the hindpaw than the control littermates (Figure 4G).

Both inflammatory pain and neuropathic pain can cause the characteristic phenomena, mechanical allodynia and thermal hyperalgesia. The CFA model and the SNI model were taken to assess the sensitivity to inflammatory pain and neuropathic pain, respectively. The hindpaws of *Tlx3cko* and control mice seriously swelled after the intraplantar injection of CFA, indicating that the inflammation occurred normally. In control mice, substantially decreased mechanical and thermal thresholds were detected after the CFA injection, indicating the existence of pain hypersensitivity. In contrast, the mechanical and thermal thresholds remained almost unchanged in *Tlx3cko* mice (Figures 4H,I), demonstrating the deficit of inflammatory pain. After the SNI surgery, the substantially decreased mechanical thresholds were detected in both groups, indicating a normal response to neuropathic pain in *Tlx3cko* mice (Figure 4J). Collectively, Tlx3 also played a pivotal role in regulating the responses to cold, inflammatory pain and capsaicin.

### Derepression of TRPV1 in *Tlx3cko* Mice

**Figure 5:**
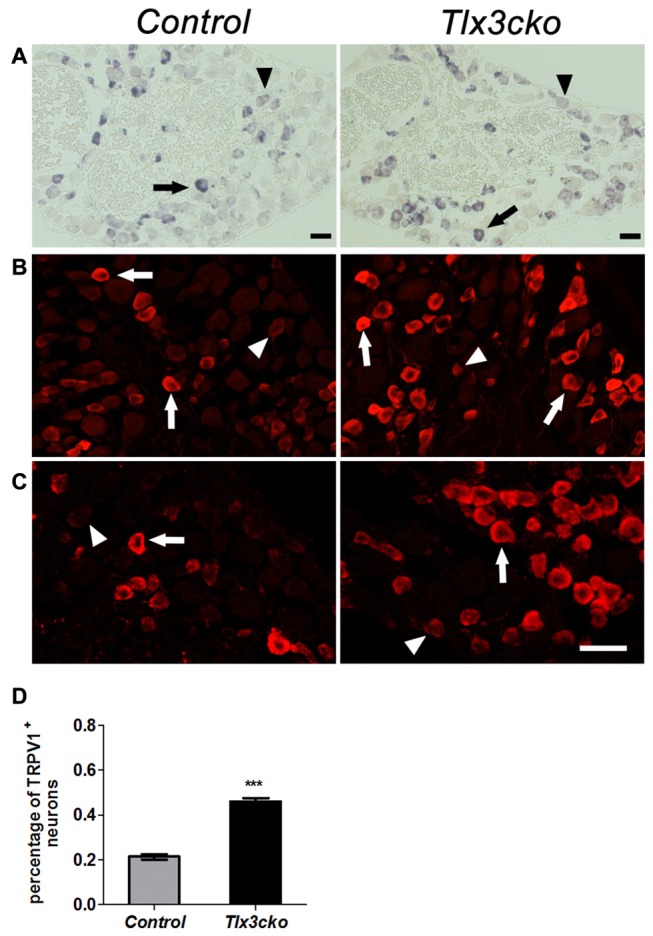
Increased expression of TRPV1 in *Tlx3cko* DRG. Immunostaining of TRPV1 protein and *in situ* hybridization of *TRPV1* mRNA on transverse sections of lumbar DRGs from adult control and *Tlx3cko* mice. Note that the percentage of TRPV1^+^ neurons was greatly increased in *Tlx3cko* mice. Arrow and arrowhead indicate the TRPV1^high^ and TRPV1^low^ neurons respectively **(A–C)**. **(A)** *In situ* hybridization signal of *TrpV1* mRNA. **(B)** Immunostaining of TRPV1 protein with antibody from Millipore Company. **(C)** Immunostaining of TRPV1 protein with antibody from Santa Cruz Company. **(D)** The statistical histograms of the percentage of TRPV1^+^ neurons in total DRG neurons in *Tlx3cko* and control mice (control, 21.3% ± 1.2%, *n* = 3; *Tlx3cko*, 46% ± 1.5%, *n* = 3; *p* = 0.0002). Scale bar represents 50 μm. ***Indicates *p* < 0.001.

The elevated pain responses evoked by capsaicin in *Tlx3cko* mice led us to examine whether the expression of TRPV1 was elevated. In DRG, TRPV1^+^ neurons can be divided into TRPV1^high^ and TRPV1^low^ neurons according to the relatively high or low expression level of TRPV1, and most of these neurons express TRPV1 at a relatively low level in wild-type mice. We previously reported that TRPV1^high^ neurons were almost completely eliminated but TRPV1^low^ neurons were unaffected in Tlx3 complete null mice at P0. In contrast, both of TRPV1^high^ and TRPV1^low^ neurons were still observed in *Tlx3cko* mice (*Tlx3* was removed around E17) at adult stages (Lopes et al., 2012), whereas the number of TRPV1^+^ neurons was not carefully quantified in our previous study. Our RNAseq data indicated that the expression level of *TRPV1* was greatly increased in *Tlx3cko* mice (mean transcript reads: control, 2263.5; *Tlx3cko*, 3572.8, *n* = 3, *p* = 1.81E-15). Consistent with our RNAseq data, we detected increased TRPV1^+^ neurons in *Tlx3cko* DRGs with *in situ* hybridization (Figure 5A). The number of TrpV1^high^ neurons was comparable between *Tlx3cko* and control mice, while the number of TRPV1^low^ neurons in mutant DRGs was approximately twice that in control DRGs. Then, we detected TRPV1 expression by IF using two kinds of antibodies at protein level and obtained consistent results. The percentage of TRPV1^+^ neurons was dramatically increased in *Tlx3cko* DRGs (control, 21.3% ± 1.2% vs.* Tlx3cko*, 46% ± 1.5%, *p* = 0.0002; Figures 5B–D). Surprisingly, the percentage of TRPV1^high^ neurons among all TRPV1^+^ cells was up-regulated from about 20% in control DRGs to 86% in *Tlx3cko* DRGs, indicating that the loss of Tlx3 function significantly increased not only the number of TRPV1^+^ neurons but also the expression level of TRPV1 at protein level.

**Figure 6:**
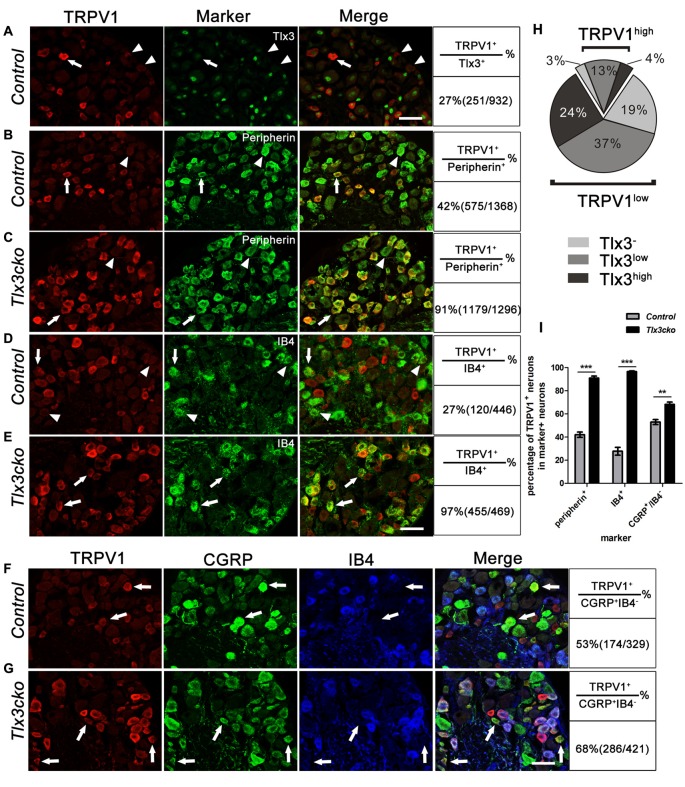
Expression pattern of TRPV1 in control and *Tlx3cko* DRG. Double immunostaining of TRPV1 and indicated markers on transverse sections of adult lumbar DRGs from control and *Tlx3cko* mice. Quantitative data were listed on the right column. **(A)** Double staining of TRPV1 and Tlx3 on transverse sections of adult lumbar wild-type DRGs. Arrow and arrowhead indicate the TRPV1^+^ neuron with a low level of Tlx3 or without Tlx3 expression. **(B,C)** Double immunostaining of TRPV1 and Peripherin. Percentage of the peripherin^+^ neurons expressing TRPV1 was significantly increased in *Tlx3cko* DRGs (control, 42% ± 2.3%, *n* = 3; *Tlx3cko*, 91% ± 1.7%, *n* = 3; *p* < 0.0001). Arrow and arrowhead indicate the Peripherin^+^ neurons with or without TRPV1 expression respectively. **(D,E)** Double immunostaining of TRPV1 and IB4. TRPV1 was expanded robustly in IB4^+^ neurons in *Tlx3cko* mice indicated by that nearly all IB4^+^ neurons expressing TRPV1, whereas TRPV1^+^/IB4^+^ neurons were occasionally observed in control mice (control, 27.7% ± 3.3%, *n* = 4; *Tlx3cko*, 96.6% ± 0.6%, *n* = 3; *p* < 0.0001). Arrow and arrowhead indicate the IB4^+^ neurons with or without TRPV1 expression respectively. **(F,G)** Triple immunostaining of TRPV1, CGRP and IB4 on transverse sections of adult lumbar DRGs. The percentage of CGRP^+^IB4^−^ neurons expressing TRPV1 was increased in *Tlx3cko* DRGs (control, 53% ± 2.1%, *n* = 3; *Tlx3cko*, 68.3% ± 2.0%, *n* = 3; *p* = 0.0062). Arrow indicates the CGRP^+^IB4^−^ neurons expressing TRPV1. **(H)** The statistic diagram of expression pattern of Tlx3 in TRPV1^+^ neuron in wild-type DRGs. **(I)** The statistical histograms of percentages of TRPV1^+^ neurons in peripherin^+^, IB4^+^ and CGRP^+^IB4^−^ neurons in *Tlx3cko* and control mice. Scale bar represents 50 μm. **, ***Indicate *p* < 0.01, *p* < 0.001, respectively.

These results suggested that Tlx3 might inhibit the expression of TRPV1 from perinatal stage. To explore this possibility, we examined whether TRPV1 and Tlx3 were expressed in distinct neurons in wild-type DRGs. As we described previously, Tlx3 was expressed broadly in DRGs, while only about 27% of Tlx3^+^ neurons expressing TRPV1 (Figure 6A). We carefully checked the Tlx3 expression in TRPV1^+^ neurons. In wild-type DRG, approximately 20% of TRPV1^+^ neurons were TRPV1^high^ neurons, 80% of TRPV1^+^ neurons were TRPV1^low^ neurons. In TRPV1^high^ neurons, the percentages of neurons with a relatively high level of Tlx3, a relatively low level of Tlx3 or without Tlx3 were 19.6%, 65.3% and 15.1%, respectively. In TRPV1^low^ neurons, the percentages were 29.6%, 46.2% and 24.2% respectively. Collectively, most of TRPV1^+^ neurons did not express Tlx3 or expressed Tlx3 at a low level (Figure 6H). Our IF data was consistent with the recently published microarray data that the transcript level of *Tlx3* was relatively high in IB4^+^SNS-cre/Tdtomato^+^ neurons, while the transcript level of *TRPV1* was relatively high in IB4^−^SNS-cre/Tdtomato^+^ cells and low in IB4^+^SNS-cre/Tdtomato^+^ neurons (Chiu et al., 2014). This mutually exclusive expression pattern of TRPV1 and Tlx3 further supported the idea that loss of Tlx3 function from the perinatal stage led to the derepression of TRPV1 in the DRG.

### Expression Pattern of TRPV1 in *Tlx3cko* Mice

To more deeply investigate how loss of Tlx3 function affects the expression pattern of TRPV1, we performed a series of co-labeling experiments with TRPV1 and other markers. Nociceptors comprise two major classes: the medium-diameter myelinated Aδ fibers and the small-diameter unmyelinated C fibers. In control DRGs, TRPV1 was limited to 42% of peripherin^+^ unmyelinated nociceptors (Figures 6B,I). In *Tlx3cko* DRGs, TRPV1 was still expressed predominantly in peripherin^+^ neurons, but the co-expression ratio increased to 91% (Figures 6C,I).

At late embryonic stages, TrkA^+^ precursors gradually segregate into IB4^+^ NP and CGRP^+^ peptidergic neurons. Our previous study indicated that loss of Tlx3 expression impaired the differentiation of IB4^+^ NP nociceptors, with a corresponding expansion of peptidergic neurons. Although the expression level of Ret in IB4^+^ neurons was markedly down-regulated in *Tlx3cko* DRGs, the numbers of IB4^+^ neurons were largely unchanged between *Tlx3cko* and control mice (Lopes et al., 2012). In control DRGs, approximately 27% of IB4^+^ neurons expressed TRPV1 (Figures 6D,I), which is consistent with a recent microarray data that *TRPV1* transcripts were enriched in IB4^−^SNS-cre/Tdtomato^+^ neurons (Chiu et al., 2014), while in *Tlx3cko* DRGs, almost all IB4^+^ neurons expressed TRPV1 (Figures 6E,I), indicating a massive expansion of TRPV1 in NP neurons. As CGRP was expanded into the IB4^+^ NP neurons in *Tlx3cko* mice, we performed triple labeling for TRPV1, IB4 and CGRP to check the expansion of TRPV1 in CGRP^+^IB4^−^ peptidergic neurons. The percentage of CGRP^+^IB4^−^ neurons expressing TRPV1 increased from approximately 53% in control littermates to approximately 68% in *Tlx3cko* mice (Figures 6F,G,I). Taken together, these results showed that loss of Tlx3 function dramatically promoted the expression of TRPV1 in IB4^+^ NP and CGRP^+^ peptidergic neurons.

**Figure 7:**
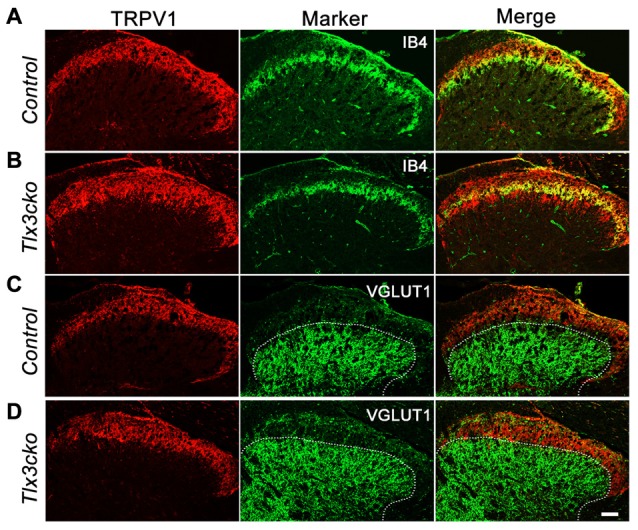
Expanded projection of TRPV1^+^ sensory fibers in spinal cord of *Tlx3cko* mice. Double immunostaining of TRPV1 and indicated markers on transverse sections of adult spinal cord. **(A,B)** Expanded projection of TRPV1^+^ sensory fibers in the spinal cord of *Tlx3cko* mice. TRPV1^+^ fibers projected to lamina I and IIo in control mice with little colocalization with IB4 **(A)**, whereas TRPV1^+^ fibers expanded to lamina IIi in *Tlx3cko* mice indicated by the colocalization of TRPV1 with IB4 **(B)**. **(C,D)** TRPV1^+^ sensory fibers were limited to lamina I and II in *Tlx3cko* mice. Though expanded, TRPV1^+^ sensory fibers were not projected to lamina III indicated by overlap of the border of TRPV1^+^ and VGLUT1^+^ fibers projection areas in *Tlx3cko* spinal cord **(D)**. The dashed line represents the dorsal border of VGLUT1^+^ neurons projection areas in spinal cords. Scale bar represents 50 μm.

The expansion of TRPV1 was further observed in the dorsal spinal cord. In the wild-type mice, CGRP^+^ peptidergic sensory afferent fibers predominantly projected to lamina I and outer lamina II (IIo) of the dorsal spinal cord, whereas IB4^+^ NP fibers mainly innervated inner lamina II (IIi) (Snider and McMahon, 1998; Chen et al., 2006). As we reported previously, the projection pattern of IB4^+^ fibers was no obvious change in *Tlx3cko* spinal cords. Here, our study showed that in control spinal cord, TRPV1^+^ fibers projected to lamina I and IIo with little colocalization with IB4^+^ fibers (Figure 7A), but in *Tlx3cko* spinal cord, the additional TRPV1^+^ fibers expanded into lamina IIi with significant colocalization with IB4^+^ fibers (Figure 7B). To identify the ventral border of TRPV1^+^ afferents in dorsal spinal cord, we co-stained the spinal cord for TRPV1 and vesicular glutamate transporter type 1 (VGLUT1). VGLUT1 was enriched in mechanoreceptors that projected mainly to lamina III-V. Little colocalization between TRPV1 and VGLUT1 was observed in *Tlx3cko* mice (Figures 7C,D), indicating that increased TRPV1^+^ primary afferents were still located in the projection area for nociceptors.

### Capsaicin Rather than Heat Activated More Spinal Neurons in *Tlx3cko* Mice

**Figure 8:**
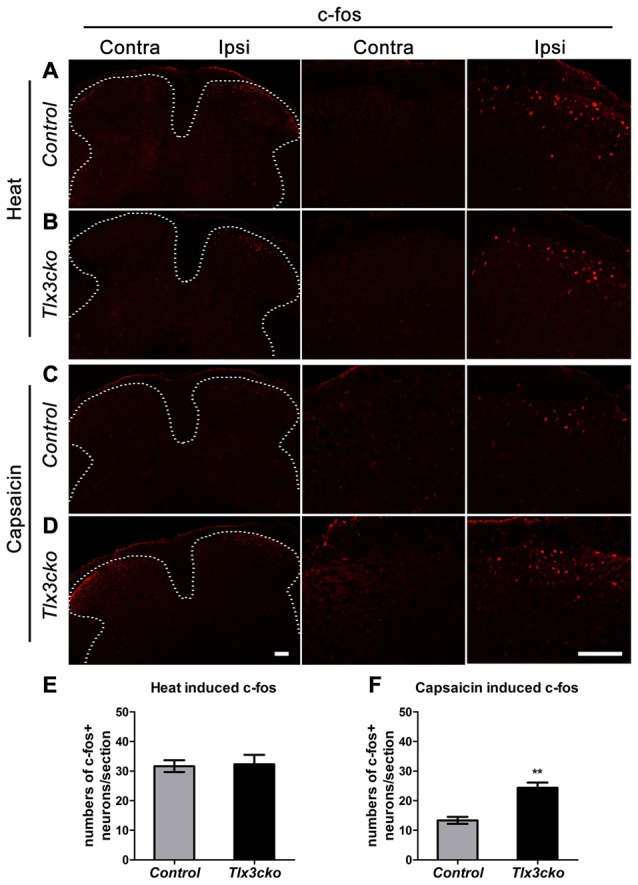
The expression of c-fos induced by capsaicin or noxious heat in spinal cords of control and *Tlx3cko* mice. Immunostaining of c-fos on transverse sections of the dorsal spinal cords after capsaicin injection or noxious heat stimulation, the images with higher magnification were shown on the middle and right column, the dashed line represents the border of gray matters **(A–D)**, the statistical data were shown on **(E,F)**. **(A,B)** Noxious heat stimulation induced comparable c-fos expression in control and *Tlx3cko* dorsal spinal cords (c-fos^+^ neuron: Ipsi control, 31 ± 2/section; *Tlx3cko*, 32 ± 3/section, *n* = 3 mice; *p* = 0.8668, 17–21 sections from each mouse were counted). **(C,D)** Capsaicin induced more c-fos expression in *Tlx3cko* spinal cords (c-fos^+^ neuron: Ipsi control, 13 ± 1/section; *Tlx3cko*, 24 ± 2/section, *n* = 3 mice; *p* = 0.0067, 15–18 sections from each mouse were counted). Note that no apparent c-fos expression was observed on contralateral spinal cords in both genotype mice after capsaicin or noxious heat stimulation. Scale bar represents 50 μm. **Indicates *p* < 0.01.

To explore whether increased TRPV1 expression would affect the primary signal input, we examined spinal neuron activation induced by capsaicin or noxious heat. The immediate early gene *c-fos* was used to identify the capsaicin- or heat-responsive neurons in the dorsal horn of the spinal cord. Consistent with the hotplate results, a 55°C water bath did not induce more c-fos expression in spinal neurons of *Tlx3cko* mice than in those of control mice (Figures 8A,B,E). However, intraplantar injection of capsaicin induced almost twice c-fos^+^ expression in *Tlx3cko* mice as in control mice (Figures 8C,D,F). Altogether, our study demonstrates that increased TRPV1 expression mainly contributed to an intense response to capsaicin in *Tlx3cko* mice, with little effect on the response to noxious heat, but we cannot rule out the possibility that loss of Tlx3 function affected the expression of other factors in the DRG for transmitting heat signals.

## Discussion

### Tlx3 Function in the DRG Is Required for Acute and Chronic Itch

Despite outstanding progresses in the characterization of the neurons and circuits for itch (Sun and Chen, 2007; Sun et al., 2009; Han et al., 2013; Bourane et al., 2015; Stantcheva et al., 2016), it remains largely unknown how the differentiation of pruriceptors and the formation of pruritic circuits are regulated at the molecular level. Xu et al. (2013) found that Tlx3 function in spinal dI5 and dIL_B_ neurons was required for the development of a subpopulation of excitatory neurons located in lamina I and II, including Sst^+^, GRP^+^ and GRPR^+^ neurons. *Tlx3^F/F^;Lbx1-Cre* mice exhibited significant deficits in multiple acute pain- and itch-related behaviors. Here, our study indicated that the function of Tlx3 in the DRG was also crucial for the differentiation of pruriceptors and itch sensation for the following reasons: (1) Tlx3 was required for the expression of a majority of itch-related receptors and neurotransmitters in the DRG; (2) acute itch induced by multiple pruritogens was greatly attenuated in *Tlx3cko* mice; and (3) chronic itch induced by dry skin was impaired in *Tlx3cko* mice.

Based on recent single-cell RNA sequencing and function studies, somatosensory neurons can be divided into distinct clusters (Chiu et al., 2014; Goswami et al., 2014; Li et al., 2016). One study classified the primary somatosensory neurons in the DRG into 11 groups (Usoskin et al., 2015). Among them, most receptors for exogenous and endogenous pruritogens are expressed in NP clusters, including NP1, NP2 and NP3. Lysophosphatidic acid receptors and MrgprD are selectively expressed in NP1 subgroups. Histamine receptors and MrgprA3 are detected in the NP2 population. NP3 clusters express various pruritic receptors and neuropeptides, including IL-31ra, Sst and Nppb. Many of these molecules are directly involved in chronic itch, including atopic dermatitis and dry skin disease. Recent studies indicated that MrgprA3^+^ and Sst^+^ neurons are a subpopulation of DRG neurons specifically responding to multiple pruritogens. Our RNAseq data revealed that loss of Tlx3 expression in Nav1.8^+^ neurons mainly inhibited the differentiation of NP1-3 and TH^+^ C-LTMRs (data not shown). In particular, most itch-related molecules specifically expressed in MrgprA3^+^ and Sst^+^ neurons were almost completely lost in *Tlx3cko* mice. Taken together, our data further revealed that Tlx3 played key roles in regulating the development of itch-related neurons in both the DRG and the spinal cord and in the formation of primary itch circuits from the periphery to the spinal cord.

### TRPV1 Pathways Are Pivotal for Chronic Itch

*Tlx3cko* mice displayed deficient dry skin-induced itch but normal ACD-induced itch. Although *Tlx3cko* mice showed significant decreases in the expression of several molecules related to chronic itch, including *Hrh1*, *IL31ra* and *MrgprA3*, obvious spontaneous scratching was still observed in *Tlx3cko* mice with dry skin or ACD, suggesting that the chronic itch was evoked by complicated factors and that the mutants retained alternative pathways for relaying chronic itch signals. Our results showed that TRPV1 was involved in these alternative pathways. A recently established model suggests that histamine evokes scratching behavior through histamine receptor-mediated activation of TRPV1 via PLCb3 by an unknown mechanism (Han et al., 2006; Imamachi et al., 2009). In *Tlx3cko* mice, although histamine-dependent acute itch was markedly attenuated, a TRPV1 antagonist still alleviated the chronic itch evoked by dry skin or ACD, suggesting that TRPV1 was required for mediating chronic itch induced by other endogenous pruritogens with distinct mechanisms. Furthermore, this model explains why hot baths and pungent food often lead to serious itch in patients with chronic itch diseases. The complicated factors and pathways involved in distinct chronic diseases reminded us that the efficacy of an anti-pruritus drug for a single target is usually limited to the management of severe chronic itch, so we speculate that it will be more effective to relieve chronic itch by systemically disturbing multiple specific targets, particularly TRPV1.

### Tlx3 Regulates Cold Pain, Inflammatory Pain and Capsaicin-Induced Pain

*Tlx3cko* mice displayed impaired cold and inflammatory pain and enhanced capsaicin-induced pain, whereas the responses to acute mechanical, noxious heat stimuli and neuropathic pain remained intact. We previously reported that Tlx3 was required for a cohort of nociceptive channels and receptors, including TRPA1, TRPM8, MrgprD, MrgprB4, and Nav1.9. As TRPM8 and TRPA1 were proved to be involved in the responses to cold and icilin (Bandell et al., 2004; Bautista et al., 2007; Colburn et al., 2007; Dhaka et al., 2007), the impaired response to cold induced by acetone evaporation and icilin in *Tlx3cko* mice was probably due to the decreased expression of TRPM8 and TRPA1.

A variety of cytokines released from immune cells and non-immune skin cells have been reported to play key roles in regulating inflammation, pain hypersensitivity and severe itch through activation their receptors, including IL31ra, Osmr, Htr1f, Cysltr2, P2rx2 and P2rx3, which were all dramatically down-regulated in *Tlx3cko* DRGs. Alternatively, it was possible that dramatic up-regulation of MrgprC11 in *Tlx3cko* mice repressed pain hypersensitivity induced by CFA-injection. Genetic deletion of Mrgpr cluster, especially MrgprC11, induced prolonged inflammatory mechanical allodynia and heat hyperalgesia compared to the wild type mice, whereas neuropathic pain remained normal (Liu et al., 2009). Injection of MrgprC11 agonists, such as BAM8-22 and JHU58, markedly inhibited calcium current in DRG neurons and further attenuated inflammatory pain hypersensitivity, suggesting that MrgprC11 might induce analgesia in persistent pain through an endogenous inhibitory pathway (Li et al., 2015).

TRPV1 is always considered a major ion channel in the response to capsaicin and noxious heat, whereas recent research reported that genetic ablation of TRPV1^+^ neurons by using TRPV1-DTR or TRPV1-DTA led to more serious heat pain deficits compared to deletion of the receptor alone (Mishra et al., 2011; Pogorzala et al., 2013), suggesting that other receptors in TRPV1^+^ neurons were also involved in responding to heat stimuli. Recently, one study demonstrated that fibroblast growth factor 13 (FGF13) selectively regulates the heat nociception by interacting with Nav1.7. The *FGF13^F/F^;Nav1.8-Cre* mice selectively lose the response to noxious heat (Yang et al., 2017). They proposed a new model for the sensing of noxious heat, which requires two steps: (1) heat stimuli activate the thermosensor, maybe TRPV1, to induce cation influx and initiate action potentials; (2) noxious heat stimuli facilitate the assembly of the FGF13/Nav1.7 complex, which can keep the action potentials firing. We examined the expression level of *FGF13* and *Nav1.7* in our RNAseq data and found that their expression levels were comparable between *Tlx3cko* and control mice (data not shown). The unchanged expression of* FGF13* and* Nav1.7* in *Tlx3cko* mice perhaps explained the normal response to noxious heat in *Tlx3cko* mice, despite of the obviously elevated TRPV1 expression.

Genetic ablation of MrgprD^+^ neurons attenuated mechanical pain responses, MrgprB4^+^ c-mechanoreceptors were associated with massage-like pleasant touch and TH^+^ C-LTMRs were sensitive to mechanical stimuli (Cavanaugh et al., 2009; Lou et al., 2013; Vrontou et al., 2013). Although the differentiation of these three group neurons was dramatically inhibited in *Tlx3cko* DRGs, the response to mechanical stimuli retained normal. Therefore, down-regulation of Tlx3-dependent receptors was not sufficient to affect the mechanical threshold.

## Author Contributions

CH designed, performed the research, analyzed the data and wrote the manuscript; FL and PL performed the research; CC analyzed data and supervised the research; ZL designed, supervised the research, analyzed the data and wrote the manuscript.

## Conflict of Interest Statement

The authors declare that the research was conducted in the absence of any commercial or financial relationships that could be construed as a potential conflict of interest.

## Abbreviations

ACD: allergic contact dermatitis
CGRP: Calcitonin Gene Related Peptide
CKO: conditional knockout
DRG: dorsal root ganglia
GPCRs: G-protein-coupled receptors
GRP: gastrin-releasing peptide
IB4: Isolectin B4
IIi: inner lamina II
IIo: outer lamina II
NP: non-peptidergic
Nppb: natriuretic polypeptide b
RNAseq: RNA sequencing
Sst: Somatostatin
VGLUT1: vesicular glutamate transporter type 1.
